# Computational speed-up of large-scale, single-cell model simulations via a fully integrated SBML-based format

**DOI:** 10.1093/bioadv/vbad039

**Published:** 2023-03-23

**Authors:** Arnab Mutsuddy, Cemal Erdem, Jonah R Huggins, Misha Salim, Daniel Cook, Nicole Hobbs, F Alex Feltus, Marc R Birtwistle

**Affiliations:** Department of Chemical and Biomolecular Engineering, Clemson University, Clemson, SC, USA; Department of Chemical and Biomolecular Engineering, Clemson University, Clemson, SC, USA; Department of Chemical and Biomolecular Engineering, Clemson University, Clemson, SC, USA; School of Computing, Clemson University, Clemson, SC, USA; SimBioSys, Inc., Chicago, IL, USA; SimBioSys, Inc., Chicago, IL, USA; SimBioSys, Inc., Chicago, IL, USA; Department of Genetics and Biochemistry, Clemson University, Clemson, SC, USA; Department of Chemical and Biomolecular Engineering, Clemson University, Clemson, SC, USA; Department of Bioengineering, Clemson University, Clemson, SC, USA

## Abstract

**Summary:**

Large-scale and whole-cell modeling has multiple challenges, including scalable model building and module communication bottlenecks (e.g. between metabolism, gene expression, signaling, etc.). We previously developed an open-source, scalable format for a large-scale mechanistic model of proliferation and death signaling dynamics, but communication bottlenecks between gene expression and protein biochemistry modules remained. Here, we developed two solutions to communication bottlenecks that speed-up simulation by ∼4-fold for hybrid stochastic-deterministic simulations and by over 100-fold for fully deterministic simulations. Fully deterministic speed-up facilitates model initialization, parameter estimation and sensitivity analysis tasks.

**Availability and implementation:**

Source code is freely available at https://github.com/birtwistlelab/SPARCED/releases/tag/v1.3.0 implemented in python, and supported on Linux, Windows and MacOS (via Docker).

Recapitulating the behavior of single cells *in silico* is a grand challenge not only for systems biology, but also for biology in general. Such an accomplishment would imply that we have a thorough understanding of all the cellular and sub-cellular processes that give rise to relevant phenotypes. Such models could enable rational engineering for biotechnology applications, or forward predictions in precision medicine (Ahn-Horst *et al.*, 2022; Mardinoglu *et al.*, 2017; Spann *et al.*, 2018; Uhlen *et al.*, 2017). Large-scale and whole-cell modeling is a suitable foundation for meeting such challenges (Goldberg *et al.*, 2018; Macklin *et al.*, 2014; Purcell *et al.*, 2013). The first such efforts focused on genome-scale metabolic modeling in multiple organisms (Patil and Nielsen, 2005; Thiele and Palsson, 2010). Subsequent efforts focused on integrating multiple ‘modules’ in addition to metabolism (e.g. gene expression, signaling, etc.) in single-celled organisms, such as *Mycoplasma genitalium*, *Escherichia coli* and *Saccharomyces cerevisiae* (Covert *et al.*, 2008; Karr *et al.*, 2012; Münzner *et al.*, 2019; Ye *et al.*, 2020), and a minimal lab-generated cell (Thornburg *et al.*, 2022), but the lack of dedicated tools specifically for large-scale/whole-cell models presented roadblocks for reuse. Algorithmic developments included rule-based modeling to specify reactions more compactly (Faeder *et al.*, 2009), and model composition tools (Gyori *et al.*, 2017; Hoops *et al.*, 2006; Lopez *et al.*, 2013; Somogyi *et al.*, 2015), but large-scale models often still presented challenges. More recent work has provided such tools like AMICI that enables SBML-specified models to be simulated quickly, PEtab (Schmiester *et al.*, 2021; Stapor *et al.*, 2018) and Datanator (Roth *et al.*, 2021) that specifies data formats for parameter estimation, formalisms that can help with unambiguous species naming (Lang *et al.*, 2020), and composition approaches, such as SBML merging (Smith *et al.*, 2015) and ours that simplify model aggregation and expansion in ways that are compatible with efficient large-scale simulation algorithms and easy to reuse (Erdem *et al.*, 2022). Not unexpectedly, however, there remains much work to be done to even technically enable large-scale and whole-cell modeling.

Here, we focused on improving communication between different modules as a major impediment for computation speed in large-scale modeling (Fig. 1). We used our recently published SPARCED model as a test case, a large-scale mechanistic model of proliferation and death signaling in single mammalian cells. This model consists of 141 genes, and 1196 unique biochemical species. It is built by translating a simple set of structured input text files into an SBML-compliant module that captures ‘protein biochemistry’ (signaling) and is simulated using AMICI, and a module that captures ‘gene expression’ using python. It can be simulated in a hybrid stochastic/deterministic mode, where gene expression dynamics follow Poisson-like processes, or a fully deterministic mode. Regardless of the mode of operation, computation speed was a major concern. Continuation of our work with the SPARCED model relies on our ability to perform more complex and resource-intensive computation, such as model initialization, parameter estimation and sensitivity analysis. Even though many such tasks require only deterministic operation, insufficient computation speed with multi-module deterministic formalism precluded further analysis, which motivated us to seek further improvement in computation speed for the general operation of the SPARCED model.

**Fig. 1.**
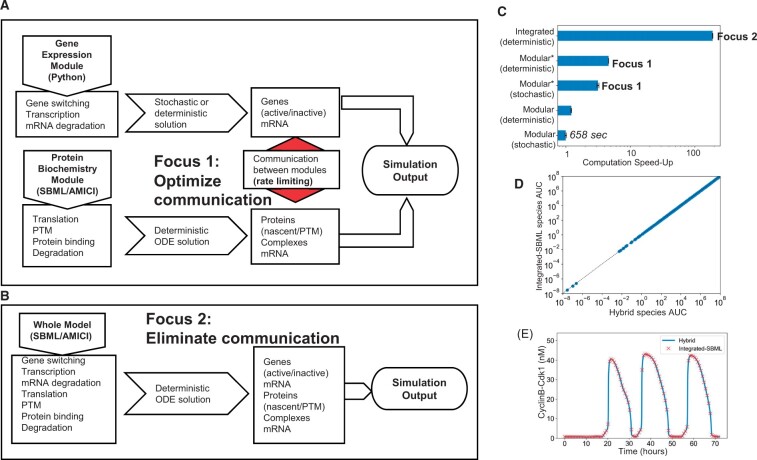
Computational speed-up of the SPARCED model. (**A**) Simulation workflow of the original SPARCED model highlighting the bottleneck of communication between the gene expression module and the protein biochemistry module. One speed-up reported here targets that bottleneck for faster stochastic simulations. (**B**) A new simulation workflow reported here that integrates the gene expression module with the protein biochemistry module using SBML, enabling large computational speed-up for deterministic simulations. No solvers yet exist for stochastic simulations at this scale. (**C**) Computation speed-up enabled by improving communication between modules (denoted by *; Focus 1) and by integrated SBML of the gene expression module with the protein biochemistry module (Integrated; Focus 2). Improving communication (Focus 1) yields 3–4 fold speed-up, and eliminating communication (Focus 2) yields ∼100-fold speed-up. A relative speed of one corresponds to 658 s. Error bars are from 10 replicate simulations. Simulations were performed on Palmetto (Clemson’s HPC resource—Intel Xeon CPU 2.5 GHz). (**D**)** **Area-under-curve for the dynamics of all model species in the original modular deterministic formulation and the integrated formulation. Simulated serum-starved MCF10A cells were treated with 1 nM EGF and 0.005 nM HGF and observed for 72 h. (**E**) An example trajectory for a biochemical correlate of cell division events from the simulations in (D) for both models, showing good agreement

As is typical for large-scale models, communication between modules was done at specified simulation time steps, in our case every 30 simulated seconds. Using Python’s built-in code profiler tool, ‘cProfile’ on our simulation code, we discovered that 94.5% of the total execution time was being spent running NumPy processes that store information in arrays. This outcome indicated a potential lack of efficiency in simulation output handling. We further sought a more detailed profiling with ‘line profiler’, which tracks execution time for every line of code. These results showed 1.4% comprised stochastic gene expression, 15.8% comprised solving ODEs (AMICI) and 81.9% comprised storing solver results into a NumPy object for inter-module communication (with the remaining 0.9% on miscellaneous overhead). We thus focused on inter-module communication as a rate limiting step for simulation speed (Fig. 1A—Focus 1).

During each 30 s time step, results from the ‘protein biochemistry’ module are saved in a ‘results’ object defined within the AMICI library. However, accessing the state matrix via the Python object interface incurred expensive reconstructions of the full NumPy array from AMICI-managed memory. These overheads could be largely avoided, since only the last column of the state matrix (corresponding to the most recent time step) was needed at each iteration. By using direct access to the SWIG pointer referencing these state variables, we were able to avoid re-reading state data, yielding a 3–4-fold simulation speed-up **(**Fig. 1C—Focus 1).

However, we reasoned that a potentially better solution to improve inter-module communication was to eliminate it altogether. This required a structural reformulation of the entire SPARCED model, whereby both modules are contained within a single SBML file. The drawback to this so-called integrated-SBML model is that no efficient numerical solvers yet exist to perform stochastic simulations on such large models. Nevertheless, fully deterministic simulations are still of use in certain situations, like model initialization by which we convert the cellular context of the model using multi-omics data (Barrette *et al.*, 2018; Bouhaddou *et al.*, 2018), parameter estimation and sensitivity analysis. In contrast, a fully deterministic simulation with the previous formulation still required communication between the ‘protein biochemistry’ ODEs, and the mean approximation of the stochastic ‘gene expression’ module, subject to inter-module communication bottlenecks. In compliance with the original SPARCED model construction workflow, we re-designed the model building pipeline to use the same set of text-based input files to output two executable models, one of which is fully SBML-specified and the other retains the native hybrid multi-module formalism. (Fig. 1B—Focus 2). After implementing this change, an over 100-fold computational speed-up was observed (Fig. 1C—Focus 2). We verified that simulation results obtained with this ‘integrated SBML’ model were identical to the original model to ensure that the reformulation of the model and its build process had not introduced any errors (Fig. 1D and E).

We next sought to examine whether this faster simulation framework provides a superior alternative to more commonly available general purpose simulation tools, such as COPASI (Hoops *et al.*, 2006). We imported the integrated-SBML model into the COPASI GUI environment. As a test case, we ran the same deterministic or hybrid stochastic simulations using both COPASI and SPARCED (serum-starved MCF10A treated with growth factors for 72 h). Both deterministic and hybrid stochastic performance were slower in COPASI (Table 1).

**Table 1.** Comparison of COPASI and SPARCED

Computer specifications: CPU: AMD Ryzen 5 5600 g (6 core) RAM: 64 GB GPU: Nvidia GTX970 OS: Ubuntu 22.04	Execution time (COPASI) (min: s)	Execution time (SPARCED) (min: s)
Deterministic		
Trial 1	06:03.13	00:01.29
Trial 2	06:03.29	00:01.31
Trial 3	06:38.61	00:01.31
Trial 4	06:03.37	00:01.37
Stochastic		
Trial 1	>20 min	01:45.58
Trial 2	>20 min	02:01.19
Trial 3	>20 min	02:04.34
Trial 4	>20 min	01:57.22

*Note*: Deterministic and stochastic simulations have been run on COPASI using the LSODA method with duration set to 259 200(s), intervals to 8640 and interval size 30(s). The COPASI Time Course deterministic simulation was run using the default settings. Integrated Reduced Model was left unchecked. Relative Tolerance was set to 1e-6. Absolute Tolerance was set to 1e-12. Max Internal Steps was set to 100 000. The Max Internal Step Size was set to 0. The COPASI Time Course hybrid simulation was run using the default settings. Max Internal Steps were set to 1 000 000. The Upper and Lower Limits were set to 1000 and 800, respectively. The Partitioning Interval was set at 1. Use Random Seed was left unchecked and random Seed was set to 1.

In conclusion, here we provide code that speeds up simulation of a large-scale model of cell behavior by ∼4-fold for stochastic simulations and ∼100-fold for deterministic simulations, by focusing on improving or eliminating communication between modules. The substantial technical improvement is predominantly impactful toward our previous work, since it will facilitate model initialization, parameter estimation and sensitivity analysis. We do not present this work as a general purpose tool for large-scale simulation; however, we believe that certain generalities in our methods and solutions may provide helpful suggestions for improvement in computational models of similar scale and structure. Namely, decreasing inter-module communication bottlenecks in stochastic and deterministic operation of large-scale models with multi-module formalism via more efficient variable handling and acceleration of fully deterministic simulation of such models by amalgamation of multiple modules into a single body mathematical description. We expect this to be impactful as a general strategy to further enable large-scale and whole-cell modeling, and also spur the development of simulation algorithms that can perform stochastic simulations using an integrated formulation.

## Funding

This work was supported by the National Institutes of Health [R35GM141891 to M.R.B.].

*Conflict of Interest*: The authors have no conflict of interest to disclose.
